# Pharmaceutical design of a delivery system for the bacteriocin lacticin 3147

**DOI:** 10.1007/s13346-021-00984-9

**Published:** 2021-04-19

**Authors:** Aoibhín Ryan, Pratikkumar Patel, Paula M. O’Connor, R. Paul Ross, Colin Hill, Sarah P. Hudson

**Affiliations:** 1 Department of Chemical Sciences, Bernal Institute, University of Limerick, Limerick, Ireland; 2 Teagasc Food Research Centre Moorepark, Fermoy Co. Cork, Fermoy, Ireland; 3 APC Microbiome Ireland Cork, Cork, Ireland; 4 School of Microbiology, University College Cork, College Road, Cork, Ireland; 5 SSPC the SFI Research Centre for Pharmaceuticals, University of Limerick, Limerick, Ireland

**Keywords:** Lacticin 3147, Bacteriocins, Physicochemical properties, Solid lipid nanoparticles, Drug delivery, Antimicrobial resistance

## Abstract

**Abstract:**

Lacticin 3147 is a dual-acting two-peptide bacteriocin which is generally active against Gram-positive bacteria, including *Listeria monocytogenes* and antimicrobial-resistant bacteria such as *Closteroides difficile* in the colon. *L. monocytogenes* infections can cause life-long effects in the elderly and vulnerable and can cause severe complications in pregnant women. *C. difficile* causes one of the most common healthcare-associated infections and can be fatal in vulnerable groups such as the elderly. Although lacticin 3147 is degraded by intestinal proteases and has poor aqueous solubility, encapsulation of the bacteriocin could enable its use as an antimicrobial for treating these bacterial infections locally in the gastrointestinal tract. Lacticin 3147 displayed activity in aqueous solutions at a range of pH values and in gastric and intestinal fluids. Exposure to trypsin and α-chymotrypsin resulted in complete inactivation, implying that lacticin 3147 should be protected from these enzymes to achieve successful local delivery to the gastrointestinal tract. The amount of lacticin 3147 dissolved, i.e. its solution concentration, in water or buffered solutions at pH 1.6 and 7.4 was low and varied with time but increased and was stabilized in gastrointestinal fluids by the phospholipid and bile salt components present. Thus, the feasibility of a solid lipid nanoparticle (SLN) delivery system for local administration of lacticin 3147 was investigated. Bacteriocin activity was observed after encapsulation and release from a lipid matrix. Moreover, activity was seen after exposure to degrading enzymes. Further optimization of SLN delivery systems could enable the successful pharmaceutical development of active lacticin 3147 as an alternative to traditional antibiotics.

**Graphical abstract:**

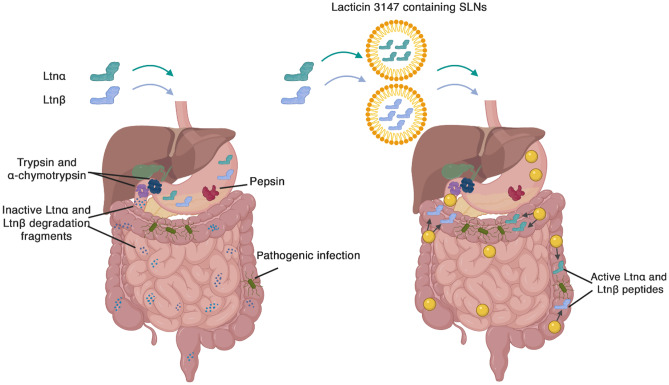

## Introduction

Antimicrobial resistance (AMR) occurs when microorganisms evolve in ways that render antibiotics ineffective. The number of bacteria becoming resistant to antimicrobial drugs is rising constantly due to non-prudent use of antibiotics and the lack of new antimicrobials coming to the market [1–3]. Consequently, AMR is endangering the effectiveness of a range of drugs that have saved millions of lives [2]. It has been projected that by 2050 there could be 10 million deaths worldwide per annum due to AMR if this crisis is not tackled, exceeding the current number of deaths from cancer [4]. Because of this, alternatives to traditional antibiotics, such as bacteriocins, are being investigated.

Bacteriocins are small, cationic, heat stable, polypeptides that are ribosomally produced by bacteria. Bacteriocins inhibit the growth of competing bacterial strains by targeting the cell membrane and/or cell wall or by targeting vital processes within the cell, including protein, DNA, and RNA metabolism [5]. The Biopharmaceutical Classification System (BCS) is used to classify small molecule active pharmaceutical ingredients (APIs) based on their aqueous solubility and permeability [6]. The knowledge of a small molecule API’s BCS class immediately leads to formulation strategies and biowaivers that can expedite their development into medicines [7]. For bacteriocins, this classification system is somewhat limited. Stability (chemical and enzymatic), solubility, unfolding, adsorption to surfaces, aggregation, and permeability are important physicochemical and biophysical factors that must be taken into consideration to enable the development of these peptides into antibiotics [8]. In general, a more in-depth classification system than the BCS is required to expedite the development of peptide biopharmaceuticals.

**Fig. 1:**
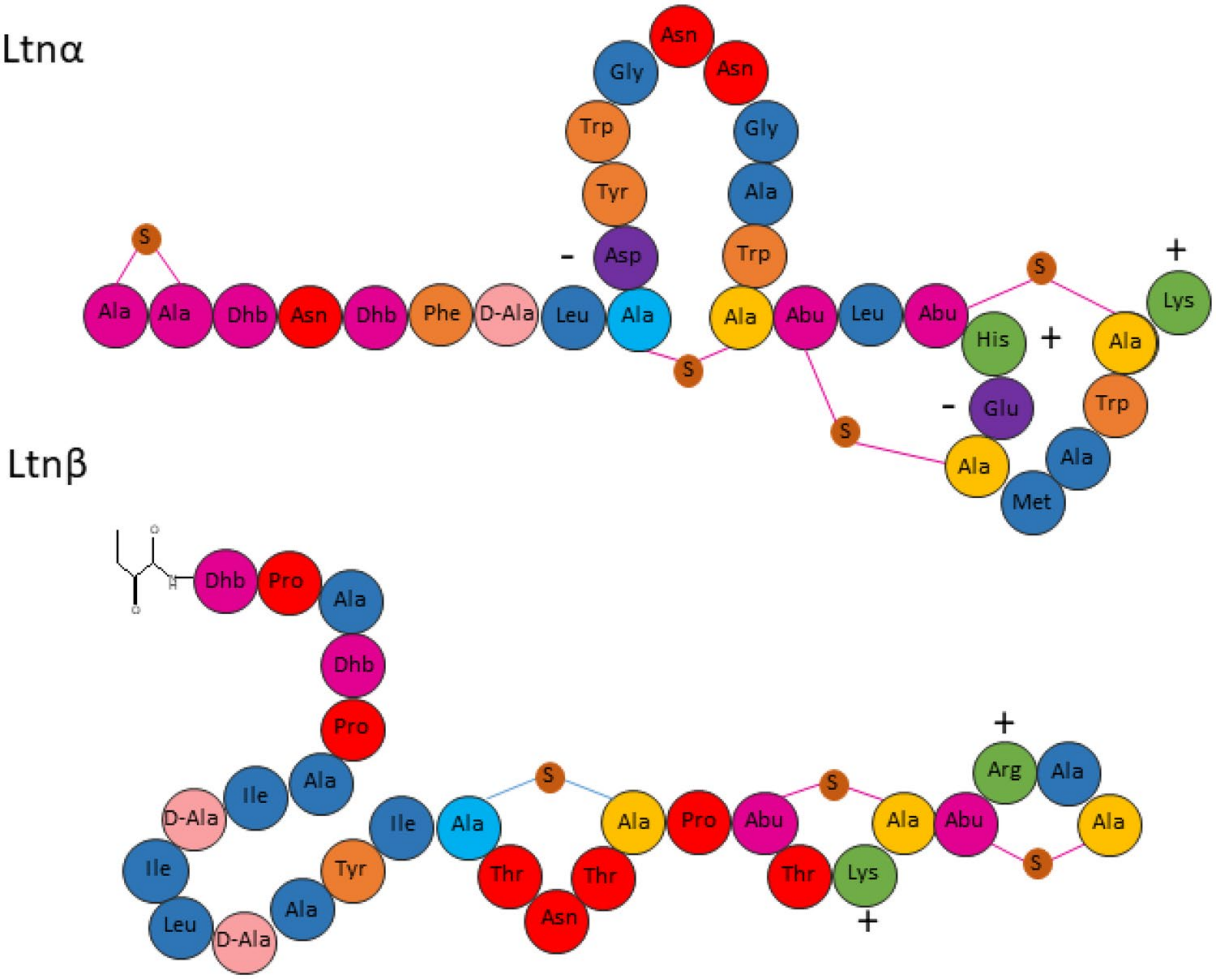
The structure of the lacticin 3147 peptides, Ltnα and Ltnβ, illustrating the D-amino acid, D-alanine, the dehydrated amino acid, dehydrobutyrine (Dhb), and Ala-S-Ala, lanthionine, and Abu-S-Ala, β-methyllanthionine rings. The positively and negatively charged residues of the peptides are also indicated by plus and minus symbols

Lacticin 3147 is a two-peptide (Ltnα and Ltnβ) bacteriocin produced by *Lactococcus lactis* DPC6577 (Fig. 1). It is classified as a lantibiotic due to the presence of lanthionine rings in its structure [9]. The two peptides act synergistically, targeting lipid II, a precursor of peptidoglycan synthesis in Gram-positive bacteria, leading to inhibition of cell wall synthesis, pore formation, and eventual cell death [10]. Ltnα exhibits low bacteriostatic activity when assayed alone, with a reported minimum inhibitory concentration required to inhibit the growth of 50% of bacteria (MIC_50_) of 200 nM against *L. lactis* HP. When equal concentrations of Ltnα and Ltnβ are added, however, the MIC_50_ drops to 7 nM indicating that the presence of both peptides at a 1:1 molar ratio allows for optimum activity [11]. Ltnβ alone has also shown bactericidal activity against *L. lactis* subspecies *cremoris* at high concentrations [12].

Lacticin 3147 is non-toxic to eukaryotic cells [13] and active at nanomolar concentrations against several clinically relevant bacteria, like *L. monocytogenes*, and antimicrobial-resistant bacteria such as *C. difficile* and methicillin-resistant *S. aureus* (MRSA) [14–16]. Approximately 223,900 *C. difficile* infections and 12,800 resulting deaths were reported in the USA in 2017 [17]. Treatment failures and recurrences of infection after the use of antibiotics are increasingly common, with a 2–38% failure rate for vancomycin and 8–50% for metronidazole [18]. A more virulent strain of *C. difficile*, PCR ribotype 027/NAP-1, which has recently been identified, is increasing the need for alternative therapies [19]. Around 323,700 clinical cases of MRSA were recorded in the USA in 2017 resulting in 10,600 deaths [17]. These figures highlight the urgent need for new treatment options for both *C. difficile* and MRSA infections. Although less threatening than *C. difficile* and MRSA, *L. monocytogenes* can be especially dangerous for pregnant women, leading to severe complications in pregnancy. It can result in lifelong health issues or death in the very young and can be fatal in elderly and immunocompromised people [20]. Oral administration of lacticin 3147 would allow for the treatment of *C. difficile* and *L. monocytogenes* infections, and its topical administration could be used to treat *S. aureus*, including MRSA, wound infections. However, the use of bacteriocins as medicines is limited due to their inherent physicochemical and biophysical properties.

Ryan et al. reported that lacticin 3147 was degraded and inactivated by trypsin and α-chymotrypsin, digestive enzymes found in the small intestines, but that its activity was unaffected by pepsin [9]. Gardiner et al., however, reported that α-chymotrypsin alone degrades lacticin 3147 [21]. Lacticin 3147 is heat stable, pH stable, and active at a neutral pH [9]. However, quantitative data on the amount of lacticin 3147 dissolved, i.e. its solution concentration, is rarely reported. In fact, only a handful of quantitative solubility studies on bacteriocins can be found in the literature [22–24], with activity studies reported in their stead. Bacteriocins display low minimum inhibitory concentrations (MICs), and thus, only low solution concentrations of peptide are required to demonstrate activity. That said, in many cases, pH effects may reduce aqueous solution concentrations to below the MIC at different physiological locations, resulting in a loss in efficacy and could also result in the precipitation of peptide aggregates in vivo that could induce an undesirable immunogenic response.

Additionally, as many proteins are administered parenterally due to low bioavailability from oral administration [25], long-acting formulations are often desirable to reduce the need for frequent administration. Long-acting formulations can be achieved using inherently slow dissolving solid phases of the therapeutic or matrices that control the release of the therapeutic via diffusion or degradation of the matrix. For example, nisin A was released from solid lipid nanoparticles (SLNs) and pediocin AcH was released from phosphatidylcholine nanovesicles with sustained release of 15–20 days and 13 days respectively [26, 27]. To design such long-acting medicines, the solubility in both aqueous and non-aqueous solvent systems is an important parameter to ensure efficient encapsulation and the desirable resulting dissolution behaviour. This study investigates the stability, solution concentration, and activity of lacticin 3147 at a range of pH values and in solutions that mimic the gastrointestinal tract (Fasted State Simulated Gastric Fluid (FASSGF) and Intestinal Fluid (FaSSIF)). We explore the suitability of lipid formulations to protect lacticin 3147 against proteases. Lacticin 3147 encapsulated in solid lipid nanoparticles shows encouraging in vitro activity compared to free lacticin 3147 suggesting their potential as an alternative local oral treatment modality to eradicate pathogenic infections in the colon.

## Materials and methods

### Materials

*L. Lactis* DPC6577 was supplied by our collaborators in Teagasc Food Research Centre, Moorepark, Cork, Ireland. M17 broth, tryptic soy broth (TSB), tryptic soy agar (TSA), XAD16N beads, β-glycerophosphate, ethanol, acetonitrile (ACN, ≥ 99.9%), isopropanol (IPA, ≥ 99.9%), methanol (MeOH, ≥ 99.9%), acetone, phosphate-buffered saline (PBS), trifluoroacetic acid (TFA, ≥ 99.9%), sodium hydroxide (NaOH), potassium chloride (KCl), sodium phosphate monobasic monohydrate, trypsin, α-chymotrypsin, pepsin, sodium taurocholate hydrate (NaTc, > 97%), L-α-phosphatidylcholine (lecithin, ∼99% purity, from bovine brain), Tween 80, and hydrochloric acid (HCl, 36.4–38%) were all purchased from Sigma-Aldrich Ireland Ltd. D-glucose, sodium chloride (NaCl), yeast extract, tryptone, magnesium sulfate heptahydrate (MgSO4.7H2O), and manganese(II) sulfate tetrahydrate (MnSO4.4H2O) were purchased from Fisher Scientific Ireland Ltd. Sinapinic acid was purchased from Bruker. Biorelevant fasted state simulated intestinal fluid (FaSSIF) powder was purchased from Biorelevant.com Ltd. Geleol™ mono and diglycerides and Transcutol® P were gift samples from Gattefossé, France. Kolliphor® RH40 was received as a gift from BASF, Germany. Deionized water was obtained from the Elga PURELAB system.

### The production and purification of lacticin 3147

Lacticin 3147 was produced and purified following a protocol developed by Rea et al. [14]. Briefly, lacticin 3147 was purified from clarified TY broth using Amberlite XAD 16 N resin, C18 Solid Phase Extraction (SPE), and finally, lacticin 3147 was separated into its individual peptides, Ltnα and Ltnβ, by reversed-phase (RP) HPLC.

### MALDI-TOF mass spectrometry

Lacticin 3147 production was confirmed by matrix-assisted laser desorption ionization-time of flight (MALDI-TOF) mass spectrometry using sinapinic acid as the matrix [28]. About 0.7 μl of the matrix solution (sinapinic acid, 4 mg/ml in 50% acetone/50% methanol) was added to the target plate and left to evaporate (30 s) at ambient conditions. Individual peptides (Ltnα or Ltnβ) were resuspended in 70% IPA and mixed at a 1:1 volume ratio with a 10-mg/ml stock solution of sinapinic acid in 60% ACN with 0.1% TFA. About 0.3 μl of this peptide-matrix solution was then added to the matrix layer previously applied to the plate and evaporated at ambient conditions. The plate was inserted into a MALDI-TOF Ultraflex mass spectrometer (Bruker) and analysed using reflectron positive 700–3500 Da mode. 100 shots were fired at 88% intensity. This method was also used for the analyses of samples from the enzyme degradation studies.

### Bioactivity of lacticin 3147 versus *L. monocytogenes* ATCC 1916

An overnight culture of *L. monocytogenes* ATCC1916 was grown in TSB media supplemented with 6 g/L yeast at 37 °C. Freeze-dried Ltnα and Ltnβ were resuspended individually in 70% IPA to make a 1-mg/ml solution and filtered through a 0.2-µm filter (polyethersulfone) PES filter. They were then diluted in sterile PBS to a final peptide concentration of 50 µg/ml. Peptide solutions were added in triplicate to a 96-well plate with sufficient PBS to give a volume of 50 µl. 150 µl of diluted bacterial cell culture (optical density (OD) of 0.1 at 595 nm) was then added to the wells. Final peptide concentrations of 150 nM (0.50 µg/ml Ltnα, 0.43 µg/ml Ltnβ), 300 nM (0.99 µg/ml Ltnα, 0.85 µg/ml Ltnβ), and 400 nM (1.31 µg/ml Ltnα, 1.14 µg/ml Ltnβ) were obtained when the wells were filled to 200 µl. Blanks were set up in triplicate with media only and PBS only. The three control wells were also set up with bacterial culture and PBS only. The 96-well plate was incubated in a Biotek ELx808 Ultra microplate reader at 37 °C for 24 h. Readings were taken at a wavelength of 590 nm every 30 min for 24 h with the plate shaken mildly before each reading.

### Preparation of buffers and fasted state simulated gastric and intestinal fluid without enzymes

Phosphate-buffered saline (1 × , pH 6.5) was prepared by dissolving one PBS tablet in 200 ml of DI water on a magnetic stirrer. After complete dissolution of the tablet, the pH of the buffer was confirmed with a pH meter. Hydrochloric acid–potassium chloride (HCl/KCl) buffer was prepared by mixing 50 ml of 200 mM KCl and 10.6 ml of 200 mM HCl in a 100-ml volumetric flask. The pH was then adjusted to pH 2.2 with 1 M NaOH, and the volume was made up to 100 mL with DI water. Fasted state simulated gastric fluid (FaSSGF) was prepared according to the method created by Vertzoni et al. [29], later amended by Bannigan et al. [30]. The final composition of the FaSSGF consisted of 80 µM NaTc, 20 µM lecithin, 9.1 mM NaCl, and 25.1 mM HCl. FaSSIF was prepared according to the instructions provided by the manufacturer (Biorelevant, London, England). It had the final composition of 3 mM taurocholate, 0.75 mM phospholipid, 148 mM sodium, 106 mM chloride, and 29 mM phosphate. The final solution was equilibrated for 2 h to stabilize the micellar particles as per manufacturer’s recommendation. FaSSGF and FaSSIF were stored for 48 h at room temperature and used within this time frame.

### Lacticin 3147′s activity in phosphate-buffered saline, FaSSIF and FaSSGF

An excess quantity of freeze-dried Ltnα and Ltnβ were resuspended individually in PBS buffer/FaSSGF/FaSSIF. The samples were then incubated for 0.5 h at 37 ± 0.5 °C with stirring at 300 rpm. At the end of the incubation period, samples were filtered through a 0.2-µm PES syringe filter and employed in the in vitro activity assay. Briefly, 25 µl of each of the filtered peptide solutions were added in triplicate to the same wells of a 96-well plate. Three control wells were set up containing 50 µl of PBS alone. 150 μl of *L. monocytogenes* ATCC 1916 with an OD of 0.1 at 595 nm was added to each well. The 96-well plate was then incubated in a Biotek ELx808 Ultra microplate reader at 37 °C for 24 h. The readings were taken as previously described.

### RP-HPLC analysis of lacticin 3147

All lacticin 3147 samples were filtered through a 0.2-µm PES filter before analysis by RP-HPLC using an Agilent 1200 Infinity Series HPLC (Agilent Technologies, USA). The HPLC separations were carried out on an Aeris 3.6 µm Peptide 100 Å 250 × 4.6 mm, liquid chromatography column with a flow rate of 0.8 ml/min, and with 0.1% trifluoroacetic acid (TFA) in water as aqueous phase-A and 0.1% TFA in acetonitrile (ACN) as organic phase-B. The total run time was 51 min. The mobile phase started with 30:70 A:B for 3 min and change linearly to 35:65 A:B over 30 min. The organic phase was then adjusted to 100% within 1 min and continued to flow for 5 min. After this, the mobile phase returned to the initial ratio to stabilize the chromatographic system. The detector was set at 214 nm. The peak area (mAU) was plotted against the concentration (1.56–500 µg/ml) to make a calibration curve. The coefficients of determination (*R*^2^ values) were 0.9963 and 0.9983 for Ltnα and Ltnβ respectively. This method was subsequently used for quantitative analysis of Ltnα and Ltnβ.

### Enzyme degradation of lacticin 3147

**Scheme 1:**
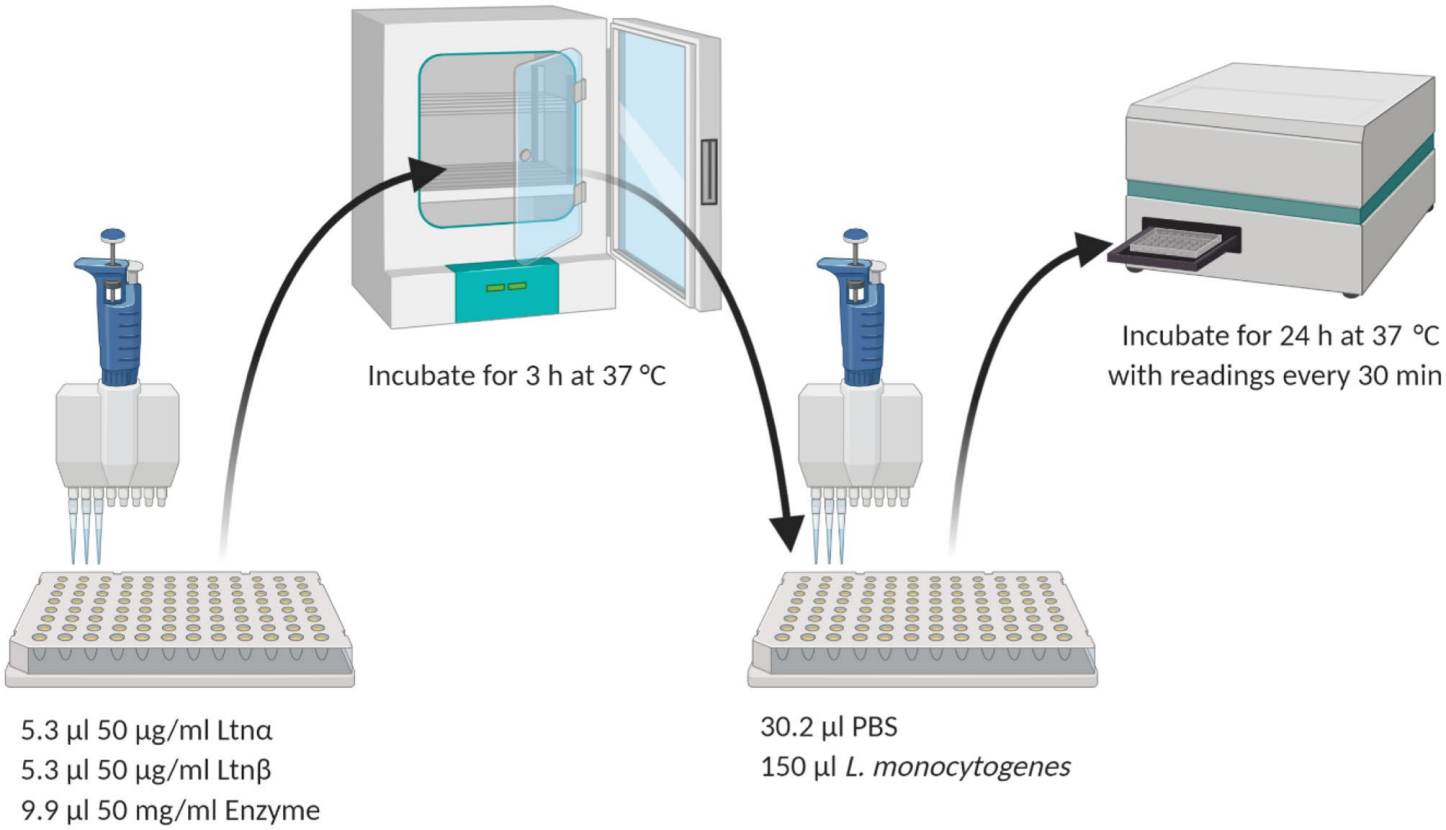
Method used to determine the effect of trypsin, α-chymotrypsin and pepsin on lacticin 3147′s activity

Trypsin and α-chymotrypsin stock solutions (50 mg/ml) were resuspended in water. Pepsin (50 mg/ml) was resuspended in 0.02 M HCL. About 1 mg/ml individual solutions of Ltnα and Ltnβ in 70% IPA were filtered and diluted (1 in 20) in sterile PBS to give a final peptide concentration of 50 µg/ml. After this, 5.3 µl Ltnα and 4.6 µl Ltnβ were added to the same well of a 96-well plate. An equal volume of enzyme was added to each well (9.9 µl), giving the molar ratios as shown in Table 1. The pH of the lacticin 3147-pepsin solution was 2.29. Control wells consisting of Ltnα and Ltnβ together, Ltnα alone, and Ltnβ alone with no enzyme were included. The 96-well plate was incubated for 3 h at 37 °C to allow the enzyme to act. After incubation, the wells were filled to 50 μl with PBS and 150 μl of diluted bacterial cell culture (*L. monocytogenes* at an OD of 0.1) was added to each well. The final peptide concentration of 400 nM (1.31 µg/ml Ltnα, 1.14 µg/ml Ltnβ) was obtained when the wells were filled to 200 µl. Blanks were set up in triplicate with media only and PBS only. The three control wells were also set up with bacterial culture and each enzyme only. The 96-well plate was incubated as previously described. The effect of the proteases pepsin, α-chymotrypsin, and trypsin on the lacticin 3147 peptides was determined by MALDI-TOF mass spectrometry. A solution containing 25 mg/ml pepsin, 13.38 µg/ml Ltnα, and 11.86 µg/ml Ltnβ was made up and left to incubate for 3 h (pH 2.29, *n* = 3). Blanks of Ltnα and Ltnβ alone and pepsin alone were included as control samples. After 3 h, 1 µl was removed from each sample and it was analysed by MALDI-TOF mass spectrometry. This was repeated for trypsin and α-chymotrypsin (Scheme 1).

**Table 1:** The molar ratio of Ltnα/Ltnβ to the digestive enzymes during the 3 h incubation period

	Molar ratio Ltnα:enzyme (nM)	Molar ratio Ltnβ:enzyme (nM)
A-chymotrypsin	1:247	1:246
Trypsin	1:265	1:264
Pepsin	1:179	1:178

Pepsin’s effect on lacticin 3147 was also investigated by RP-HPLC. A solution containing 60 mg/ml pepsin and 0.0625 mg/ml Ltnα and Ltnβ was made up and was incubated at 37 °C for 3 h. After incubation, aliquots were taken, filtered, and analysed by RP-HPLC to determine if pepsin degrades Ltnα or Ltnβ to any extent.

### The effect of pH and gastrointestinal simulated fluids on the solution concentration of lacticin 3147

Solutions of the individual lacticin 3147 peptides (500 µg/ml) were made up in PBS (pH 7.4), HCl/KCl buffer (pH 2.2), FaSSGF (pH 1.6), FaSSGF without lecithin, FaSSGF without NaTc, and FaSSIF (pH 6.5). The solutions were stirred at 300 rpm at 37 °C for 0.5 h, then placed in a shaking incubator at 60 rpm and 37 °C. At 0.5 h, 5 h, and 24 h, 100 µl aliquots of each sample were taken and analysed by RP-HPLC. Samples were only taken at 0.5 h and 24 h for lacticin 3147 in FaSSGF without lecithin and FaSSGF without NaTc.

### Micelle characterization

Solutions of the individual lacticin 3147 peptides (500 µg/ml) were prepared in FaSSIF, stirred at 300 rpm at 37 °C for 0.5 h. At 0.5 h, a 1-ml aliquot was taken and filtered through a 0.2-µm PES filter. It was analysed for micelle formation through dynamic light scattering (DLS) using the Malvern Nanozetasizer and a disposable plastic cuvette. The material selected was lipid (refractive index 1.5), and the dispersant was water (refractive index 1.33). The zeta potential was also determined using the same instrument and a disposable folded capillary cell.

### Encapsulation of lacticin 3147 into solid lipid nanoparticles

#### Preparation of the lacticin 3147 loaded SLNs

SLNs were prepared using the microemulsion template technique reported in the literature [31]. About 3–6 mg of the individual lacticin 3147 peptides were solubilized in a mixture of lipid phase (0.055 g Geleol™- 10% Lecithin) maintained at a temperature above its melting point (65 °C) with a surfactant-cosurfactant phase (0.25 g Kolliphore® RH 40:Transcutol® P, 2:1) and DI water (0.7 ml). This formed a warm preconcentrate microemulsion which was sonicated for 3 min and then diluted in 5 ml cold water (2–8 °C) under stirring at 1000–1200 rpm for 5–7 min to form the SLNs dispersion (approximately 6 ml). The SLN solution was then filtered to remove any precipitated lacticin 3147. Similarly, blank SLNs were also prepared without adding lacticin 3147.

#### Particle size distribution and zeta potential analysis of lacticin 3147 loaded SLNs

The particle size and poly diversity index (PDI) of the SLNs were measured by photon correlation spectroscopy, using a Nanozetasizer (Malvern Instruments Ltd., Worcestershire, UK). Samples were diluted 50 times using DI water to guarantee that the dispersion was within the instrument’s ‘dynamic light scattering intensity’ sensitivity range of light. The zeta potential of the concentrated solutions was also determined using the Nanozetasizer. Measurements were carried out in triplicate at 25 °C. The refractive indexes selected were 1.5 for the dispersed phase (lipid) and 1.33 for the dispersant (water).

#### Determination of total peptide content (TP), free peptide content (FP), and encapsulation efficiency (EE%) of lacticin 3147 loaded SLNs

To determine the total peptide content, lacticin 3147 loaded SLN dispersions were diluted with methanol, vortexed, and sonicated for 5 min. The samples were filtered using a 0.2-µm PES syringe filter and analysed by RP-HPLC as discussed in ‘RP-HPLC analysis of lacticin 3147′. To establish the free peptide content of the SLN dispersions, they must be destabilized. This was done by adding 200 mg of NaCl to 0.5 ml of the dispersions followed by vortexing, then centrifugation for 10 min at 10,000 rpm. This separated the lipid (α-SLN and β-SLN) and aqueous phases. The destabilized SLN dispersion containing tube was carefully removed from the centrifuge and placed in the fridge for 10 min to allow the lipid layer to harden. Using a needle, the clear supernatant aqueous layer was removed, diluted with IPA, and centrifuged again. It was then filtered using a 0.2-µm PES filter and analysed by RP-HPLC to detect the free peptide content. The concentration of encapsulated Ltnα and Ltnβ was determined by subtracting the free peptide content from total peptide content. The encapsulated efficiency of Ltnα and Ltnβ was calculated using the following calculation:

$$\mathrm{Encapsulation}\;\mathrm{efficiency}(\%)=\frac{\mathrm{total}\;\mathrm{peptide}-\mathrm{free}\;\mathrm{peptide}}{\mathrm{total}\;\mathrm{peptide}}\times100$$

#### Activity of lacticin 3147 SLNs vs free peptide

The activity of the lacticin 3147 SLNs was evaluated using the well diffusion assay method [32], on TSA supplemented with 6 g/L yeast. The inhibition zones were reported in millimetre (mm) using electronic Vernier callipers. Briefly, TSA plates were inoculated with 200 µl *L. monocytogenes* ATCC1916 (OD of 0.1 at 595 nm) under aseptic conditions. Wells were bored in the agar after it hardened using a sterile 6-mm cork borer. α-SLN and β-SLN were filtered using a 0.2-µm PES filter (SLNs can pass through), and 12.5 µL of each was added to the same well. The blank SLNs were used as negative controls (25 µL). Ltnα (12.5 µL) and Ltnβ (12.5 µL) solutions were used as positive controls. Free Ltnα and Ltnβ solutions with the same concentration as the TP content of the lacticin 3147 SLNs were suspended in water, vortexed, stirred for 5 min at 1100 rpm, and filtered to remove any undissolved lacticin 3147. The free peptides were filtered again using a 0.2-µm PES filter before addition to the wells to ensure sterility. All tests were performed in triplicate.

#### Activity of lacticin 3147 SLNs after incubation with α-chymotrypsin vs free peptide

α-SLN and β-SLN were filtered using a 0.2-µm PES filter, and 25 µl of each were added to the same well of a 96-well plate. About 50 µl of a 50 mg/ml solution of α-chymotrypsin was added to these wells. A blank was set up with 50-µl water instead of α-chymotrypsin. This was repeated for free peptide solutions prepared as outlined above. The 96-well plate was incubated for 3 h at 37 °C. The 96-well plate was then removed from the incubator, and 25 µl of each solution was added in triplicate to 6-mm wells on TSA (6 g/L yeast) plates inoculated with 200 µl *L. monocytogenes* ATCC1916. The plates were incubated at 37 °C for 24 h, after which the inhibition zones were measured using an electronic Vernier callipers.

## Results and discussion

### Bioactivity of lacticin 3147

**Fig. 2:**
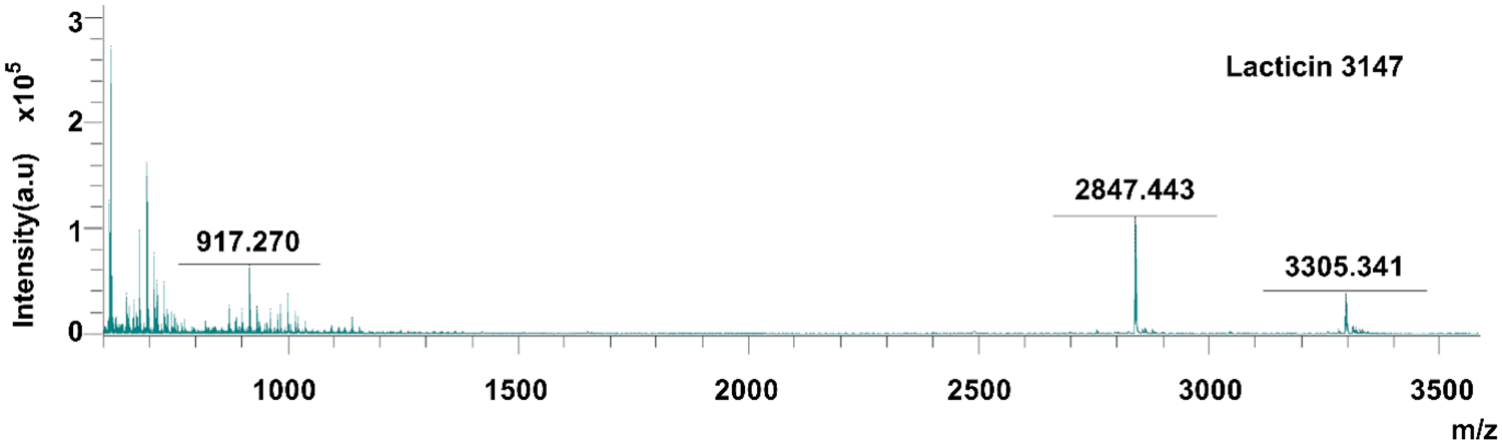
MALDI-TOF mass spectra of lacticin 3147

**Fig. 3:**
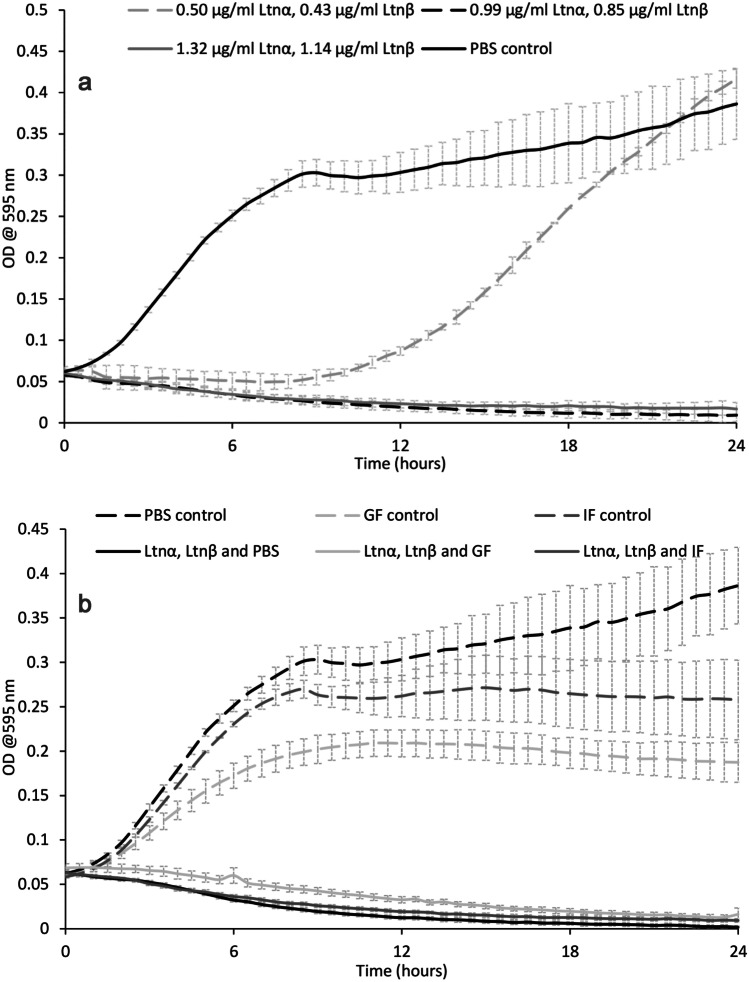
The bioactivity of lacticin 3147 against *L. monocytogenes* ATCC 1916 when **a)** dissolved in 70% IPA, filtered, and then diluted in PBS and **b)** resuspended in excess in PBS, FaSSGF, and FaSSIF and then filtered

Successful production and purification of lacticin 3147 from *L. lactis* DPC6577 were confirmed by MALDI-TOF mass spectrometry with masses of 3305.341 Da and 2847.443 Da recorded for Ltnα and Ltnβ respectively (Fig. 2). These masses correspond with those previously reported in the literature [28]. Lacticin 3147′s activity against various strains including *L. monocytogenes *and* C. difficile* has been previously reported in the literature [11, 14–16, 33, 34]; however, for the purpose of this study, *L. monocytogenes* was used to establish the impact of solution composition, pH, and process parameters on its activity. Our studies found that lacticin 3147 fully inhibited *L. monocytogenes* ATCC1916 at a concentration as low as 0.99 µg/ml Ltnα and 0.85 µg/ml Ltnβ (300 nM) (Fig. 3a) via growth curve assays. Lacticin 3147 was also active against *L. monocytogenes* ATCC 1916 when filtered (to remove any undissolved lacticin 3147) after addition in excess to PBS (pH 7.4), FaSSGF (pH 1.6), and FaSSIF (pH 6.5) (Fig. 3b). This implies more than 0.99 and 0.85 µg/ml, of Ltnα and Ltnβ respectively dissolved in each of these solutions. Ryan et al. reported that lacticin 3147 is active against *L. lactis* subsp. *cremoris* HP at a neutral pH [9]. Quantitative solution concentration data is important as dissolved lacticin 3147 is required for activity and for systemic distribution (if desired) in vivo. Lacticin 3147 precipitates/aggregates could also provoke an unwanted immune response in patients. The components of the media used during in vitro activity assays may be necessary for solubilization of the peptide masking solubility challenges that will occur in vivo. The amount of lacticin 3147 dissolved in, for example, an aqueous-based buffer such as PBS, or simulated bodily fluids such as gastrointestinal media, and the stability of these solutions in terms of precipitation from solution, has not previously been reported.

### Enzymatic degradation of lacticin 3147

**Fig. 4:**
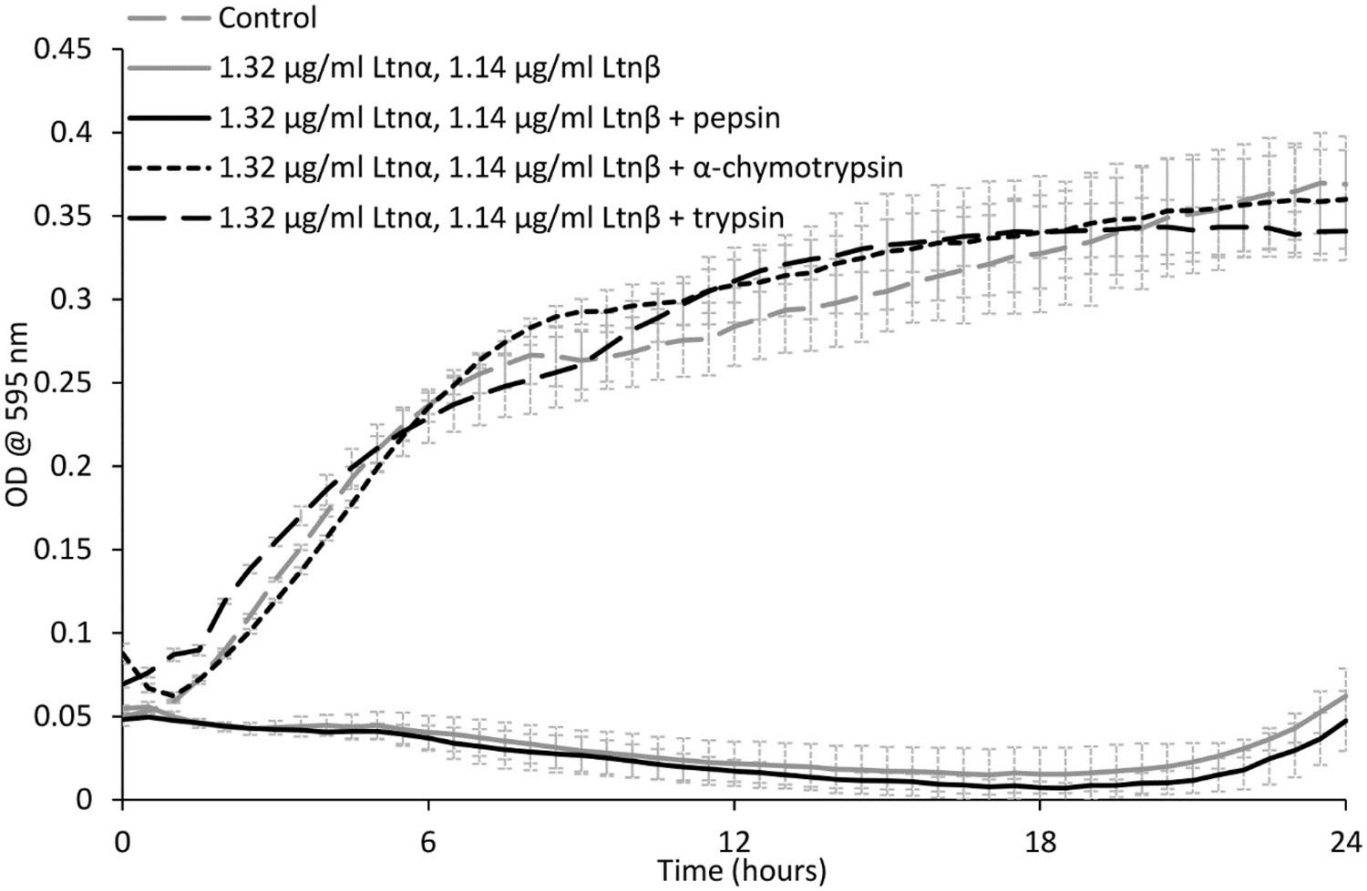
The effect of 25 mg/ml pepsin, α-chymotrypsin, and trypsin on lacticin 3147′s activity against *L. monocytogenes* ATCC 1916

A study on the effect of the proteases α-chymotrypsin, trypsin, and pepsin on the activity of lacticin 3147 was carried out (Fig. 4).

After 3-h incubation with pepsin, lacticin 3147 remains active and no increase in bacterial growth compared to lacticin 3147 alone can be seen (Fig. 4). This confirmed that pepsin does not degrade either lacticin 3147 peptide as previously determined by Ryan et al. and Gardiner et al. [9, 21]. When Ltnα and Ltnβ were incubated with trypsin and α-chymotrypsin, a growth curve similar to that of the bacterial control can be observed implying that both α-chymotrypsin and trypsin degrade at least one of the lacticin 3147 peptides.

**Fig. 5:**
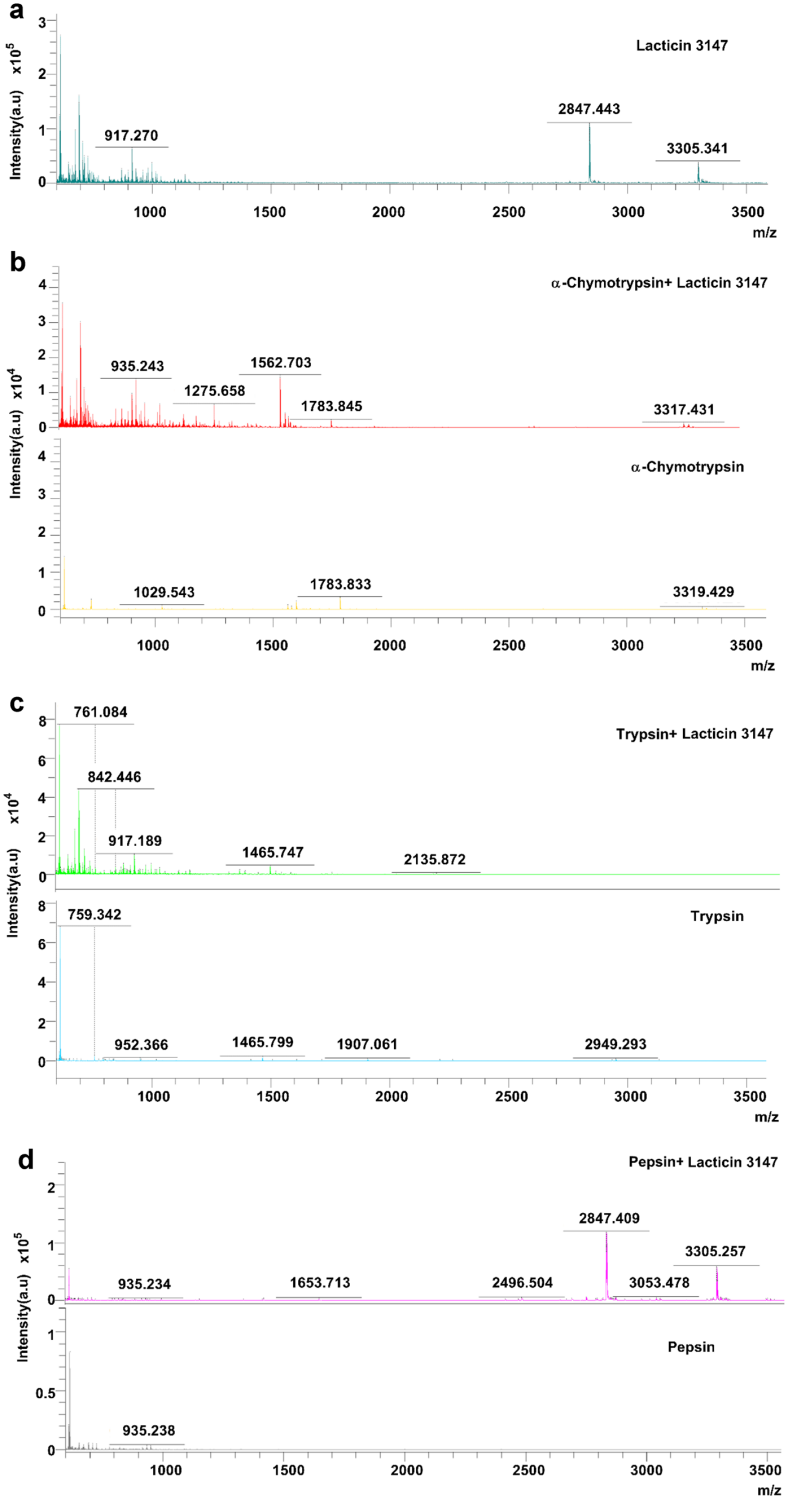
MALDI-TOF mass spectra of lacticin 3147 incubated for 3 h with **a)** water, **b)** α-chymotrypsin, **c)** trypsin, and **d)** pepsin and compared to an α-chymotrypsin/trypsin/pepsin blank

Subsequently, MALDI-TOF mass spectrometry on the peptides-enzyme solutions after 3 h of incubation was performed (Fig. 5). In the α-chymotrypsin-lacticin 3147 and trypsin-lacticin 3147 samples, no masses corresponding to intact Ltnα or Ltnβ can be observed (Fig. 5b and c), confirming that the absence of activity for these solutions in Fig. 4 is due to the degradation of both lacticin 3147 peptides by α-chymotrypsin and trypsin. Masses corresponding to Ltnα and Ltnβ can be seen in the pepsin-lacticin 3147 spectrum, verifying that neither lacticin 3147 peptide was degraded by pepsin.

**Fig. 6:**
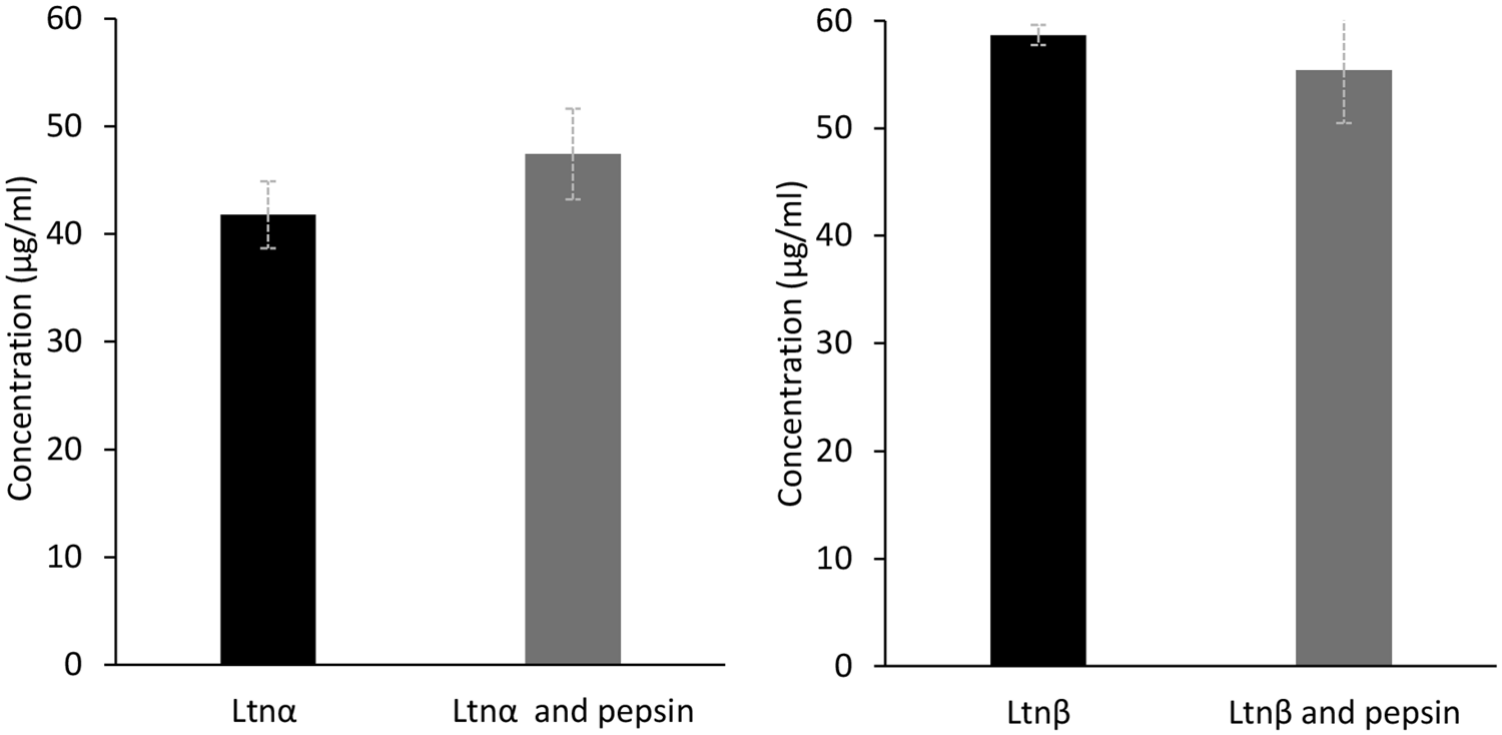
Concentration of the individual lacticin 3147 peptides (approximately 62.5 µg/ml) with and without pepsin (60 mg/ml)

As MALDI-TOF analysis can only tell us if the peptide is present in the sample or not and does not give the concentration of Ltnα and Ltnβ in solution after exposure to pepsin, RP-HPLC analysis was carried out on the peptide-pepsin solutions (Fig. 6). It is important to note that the ratio of peptide (62.5 µg/ml Ltnα/Ltnβ) to pepsin (60 mg/ml pepsin) used here is not the same as that used for the activity test or the MALDI-TOF analysis. A higher concentration of Ltnα/Ltnβ was required to allow for the detection of the lacticin 3147 peptides by RP-HPLC to quantify any degradation if it is occurring. This was not repeated for α-chymotrypsin or trypsin as the lacticin 3147 peptides were shown to be fully degraded by these enzymes via MALDI-TOF mass spectrometry analysis (Fig. 5b and c).

The concentrations of Ltnα and Ltnβ with and without pepsin are not significantly different (Fig. 6). This shows that neither peptide is degraded by pepsin to any detectable extent despite the presence of adjacent hydrophobic residues, which are targeted by pepsin, in Ltnα (Y11 and W12). The location of these target amino acids, in Ltnα’s lanthionine B ring, may shield them from the active site of pepsin. This is likely as rings A, C, and D were found to protect Ltnα from enzyme degradation by Suda et al. though ring B was not tested [35].

### The effect of pH and biorelevant media on the solution concentration of lacticin 3147

Lacticin 3147 is active at very low concentrations. To quantify solution concentrations and monitor solution behaviour, a RP-HPLC method was developed. The lowest concentration detected, i.e. the limit of detection (LOD) of this method, was 1.56 µg/ml. It is important to note that the concentration required for activity, 0.99 µg/ml Ltnα and 0.85 µg/ml Ltnβ, is below the HPLC LOD. The consequence of this is that lacticin 3147 may not be detectable in solution by HPLC, but there may still be enough dissolved to show activity.

**Fig. 7:**
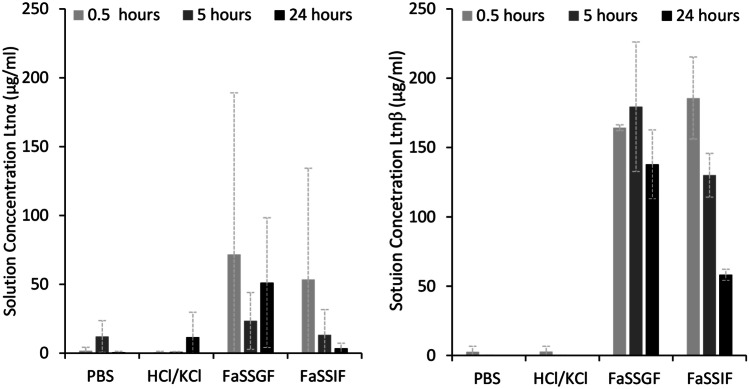
Solution concentrations of Ltnα (left *n* = 3) and Ltnβ (right *n* = 2, FaSSIF *n* = 3) after addition of 500 µg/ml peptide in PBS buffer, HCl/KCl buffer, FaSSGF, and FaSSIF at 37 °C with stirring at 300 rpm for the first 0.5 h and subsequent shaking at 60 rpm at 37 °C followed by filtration and HPLC analysis

Ltnα and Ltnβ’s solution concentration at different pH and in biorelevant media was measured. The results of this study can be seen in Fig. 7.

A low and variable concentration of dissolved Ltnα and Ltnβ can just be detected in solution in PBS buffer and HCl/KCl buffer. No 24-h time point was taken for Ltnβ in either of the buffer solutions due to the absence of a peak on the HPLC chromatogram at the 5-h time point, indicating that there was no detectable peptide in solution. It is not surprising that Ltnβ has a lower aqueous solution concentration than Ltnα as it is the more hydrophobic peptide, requiring a higher % ACN to elute it from the RP-HPLC column during purification. Concentrations of 1.7 ± 2.6 µg/ml (Ltnα) and 2.8 ± 3.9 µg/ml (Ltnβ) were detected in solution in PBS at 0.5 h, almost double and triple the reported MIC required for Ltnα and Ltnβ respectively, although large standard deviations indicate the instability of the solution. This implies that for an intravenous aqueous injection of more than double the reported MIC of lacticin 3147, solubilizers would be needed to stabilize the solution and prevent precipitation of the peptide in vivo. It also implies that PBS may not be a suitable storage buffer for lacticin 3147.

Ltnα and Ltnβ have isoelectric points (pIs) of 5.32 and 8.65 respectively [36]. Thus, it was expected that Ltnα and Ltnβ’s solution concentration would increase in a pH 2.2 (KCl/HCl) solution compared to a pH 7.4 (PBS) solution as both peptides would be further away from their isoelectric point (with a larger positive surface charge) at pH 2.2. The highest concentration of dissolved Ltnα detected at pH 2.2 (11.6 ± 18.1 µg/ml at 24 h) was not significantly different from the highest concentration of dissolved Ltnα observed in PBS (12.1 ± 11.5 µg/ml at 5 h). The solution concentration of Ltnβ in PBS and the KCl/HCl buffers at 0.5 h are also not significantly different, 2.8 ± 3.9 µg/ml and 2.8 ± 2.0 µg/ml respectively.

Ltnα initially dissolved in FaSSGF and FaSSIF (71.8 ± 117.3 µg/ml and 53.5 ± 80.8 µg/ml respectively), but these solution concentrations were extremely unstable and showed large standard deviations. The concentration of Ltnβ in FaSSGF and FaSSIF at all time points is higher and more stable than that of Ltnα and also significantly higher than its concentration in PBS or KCl/HCl buffer. About 164.3 ± 2.1 µg/ml of Ltnβ was found to dissolve in FaSSGF (pH 1.6) initially. and it did not change significantly over 24 h (Fig. 7). From an approximately 500 µg/ml solution of Ltnβ in FaSSIF, almost 185.6 ± 29.6 µg/ml was found to be in solution initially and decreased to 58.2 ± 4.0 µg/ml at 24 h. These results indicate that the components of the simulated gastrointestinal fluids solubilize both lacticin 3147 peptides, with a larger solubilising and stabilising effect on Ltnβ than Ltnα.

**Fig. 8:**
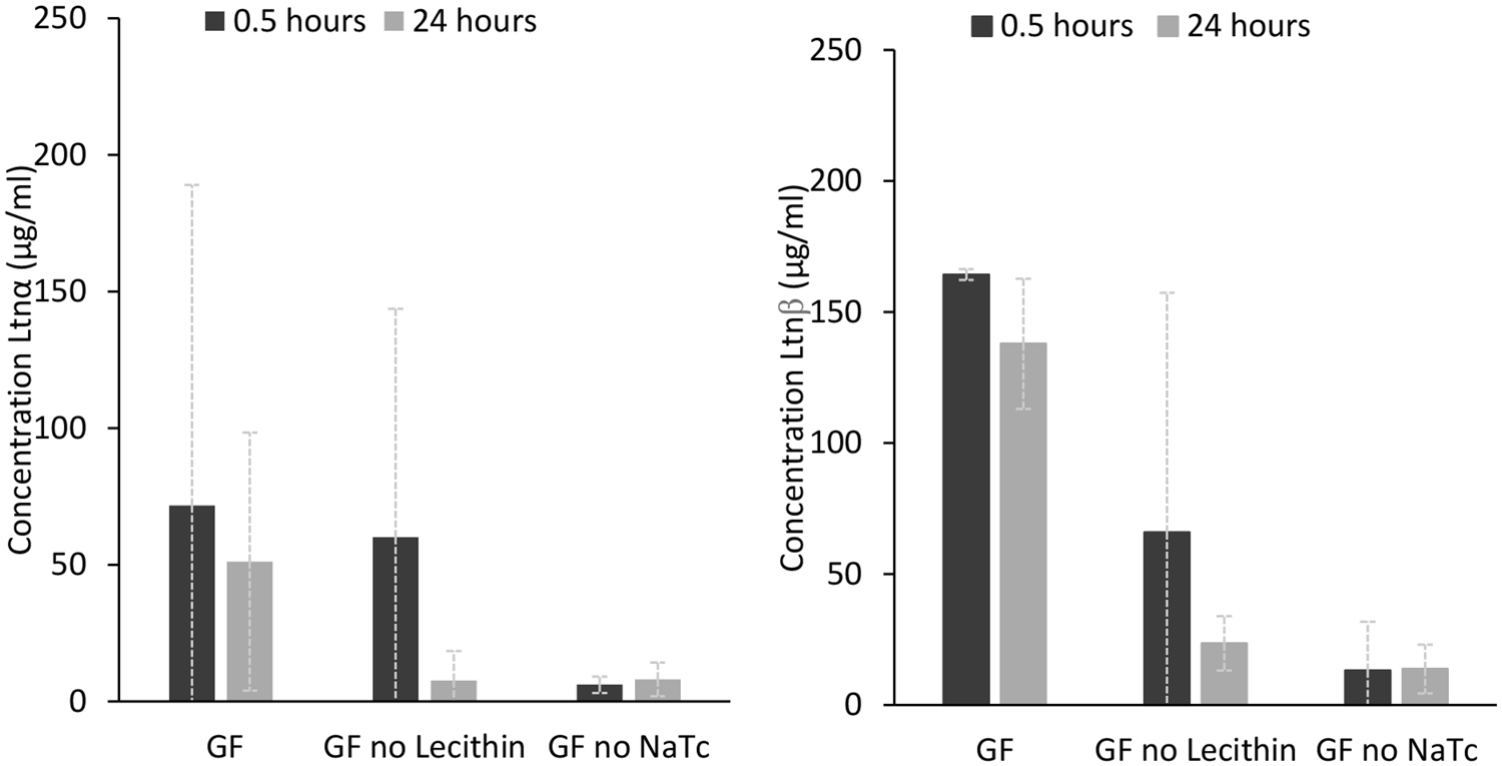
Solution concentrations (µg/ml) of Ltnα (left) and Ltnβ (right) after addition of 500 µg/ml peptide in FaSSGF (GF), GF without lecithin, and GF without NaTc at 37 °C with stirring at 300 rpm for the first 0.5 h and subsequent shaking at 60 rpm

The concentration of NaTc in FaSSGF, 80 µM, is below the critical micelle concentration (CMC) value for a 4:1 NaTc:lecithin solution, 250 µM [37]. Thus, encapsulation into micelles can be ruled out as the cause for increased lacticin 3147 solubility in FaSSGF. However, Ltnα and Ltnβ must be interacting with the lecithin and NaTc molecules present, resulting in an increase in their solution concentration, since a higher concentration of Ltnα and Ltnβ in solution was observed in FaSSGF compared to in the HCl/KCl pH 2 buffer. The solution concentration of Ltnα and Ltnβ in FaSSGF without lecithin and without NaTc was investigated to determine the effect of the individual substances on the solution concentration of lacticin 3147 (Fig. 8).

From the above figure, it can be deduced that NaTc causes an increase in the solution concentration of Ltnα and Ltnβ but that lecithin is also required to keep Ltnβ in solution.

The concentration of NaTc in FaSSIF, 3 mM, is above the CMC value for a 4:1 NaTc:phospholipid solution, 0.25 mM [37]. Because of this, it is feasible that the increase in the concentration of dissolved Ltnα and Ltnβ in FaSSIF could be due to their entrapment into micelles. Ltnβ was more soluble and remains dissolved in the FaSSIF solution longer than Ltnα (Fig. 7). The entrapment of molecules into micelles relies on hydrophobic interactions between the hydrophobic tails of the lipids and the molecule. Therefore, the higher hydrophobicity of Ltnβ could account for its higher concentration in solution. Ltnβ is also positively charged at pH 6.5. This would also allow it to bind to the negatively charged head groups of the micelles or individual phospholipid and taurochlorate molecules, thus increasing the amount of Ltnβ dissolved in solution. Ltnα is negatively charged at pH 6.5 and therefore, would be repelled from the head groups, and only encapsulation into the hydrophobic core of the micelles would increase its solution concentration.

**Fig. 9:**
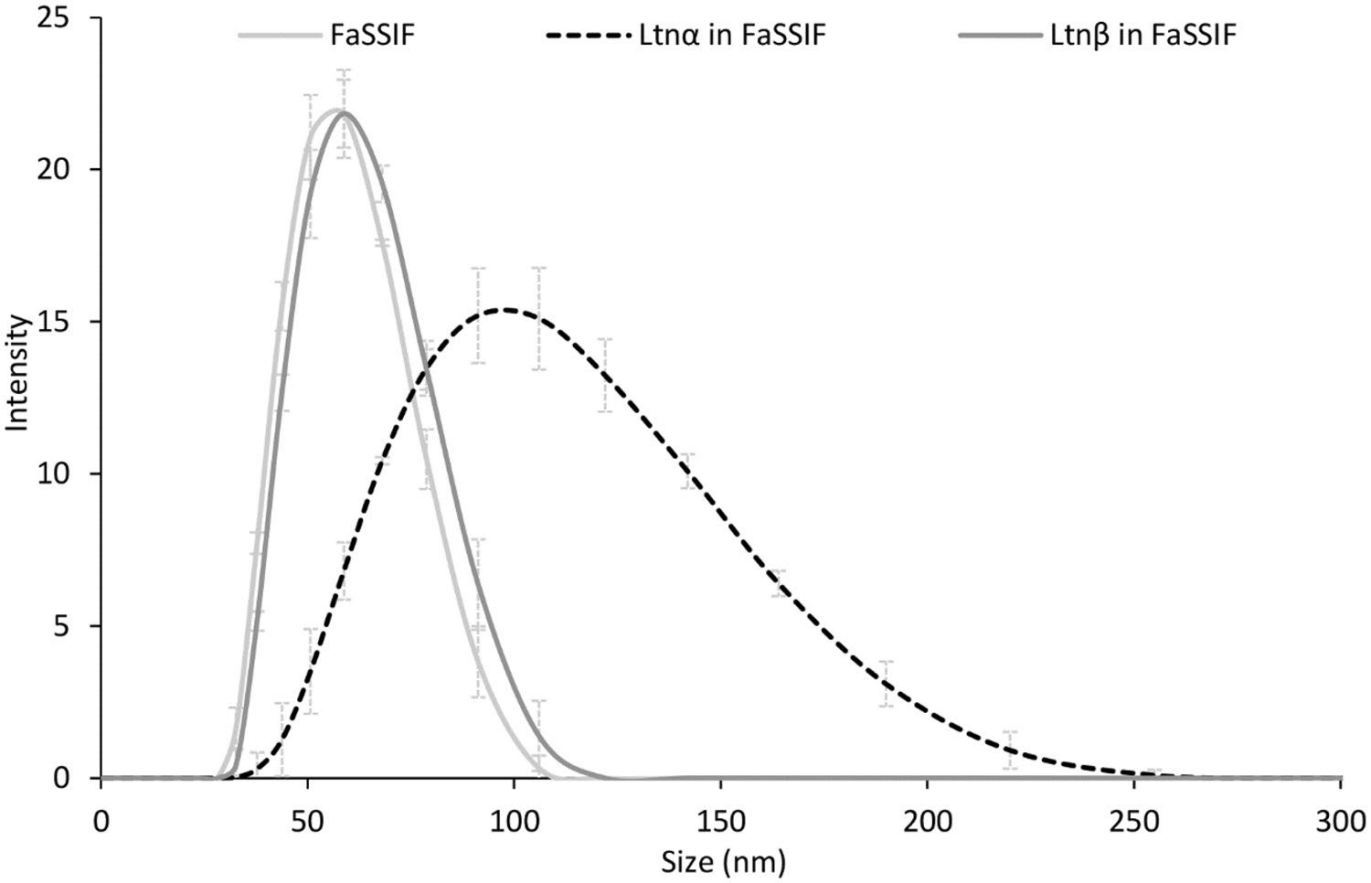
Measurement of micelle size distribution in FaSSIF in the absence or presence of Ltnα or Ltnβ at 0.5 h

To determine the presence of micelles in solution, FaSSIF was analysed by dynamic light scattering (DLS) with a zeta sizer in the nano range (Fig. 9). A peak was seen at 53 nm indicating that NaTc/phospholipid micelles are present in the FaSSIF solution. An increase in size of the micelles was observed in the FaSSIF solutions containing the lacticin 3147 peptides. This indicates the inclusion of both Ltnα and Ltnβ into the micelle structure, although a larger increase in size with Ltnα was observed. This could perhaps be due to the repulsion of the negatively charged Ltnα from the charged head groups of the phospholipid and taurochlorate molecules and predominant encapsulation inside the hydrophobic core of the micelles. Zeta potential measurements were conducted to probe whether the Ltnα or Ltnβ interact with the ionic head groups of the micelles. The results showed an increase of zeta potential of + 3.3 and + 5.5 mV for FaSSIF containing Ltnα and Ltnβ respectively compared to FaSSIF alone after 24 h. This indicated that while Ltnα appears to be encapsulated into the core of the micelles, increasing their size, the more favourable positive charge on Ltnβ may increase binding to the micelle surface or individual phospholipid and taurochlorate molecules by electrostatic interactions, leading to a greater concentration of Ltnβ in solution overall.

### Solid lipid nanoparticles as a formulation strategy for the delivery of lacticin 3147

The low and unstable aqueous solubilities of Ltnα and Ltnβ in PBS, Ltnα in FaSSGF and FaSSIF, and to a lesser degree, Ltnβ in FaSSGF and FaSSIF, mean that formulation strategies will be needed for all routes of administration of lacticin 3147. In PBS, solution concentrations only reached approximately double the required MIC against *L. monocytogenes* ATCC 1916 and these concentrations were unstable over time. This would be a cause for concern for parenteral administration [8]. At solution pH values below their pI, the solubility of neither lacticin 3147 peptide improved. Thus, parenteral administration of lacticin 3147 solutions will require solubilising and stabilising additives to prevent aggregation and potential immunogenic responses in vivo. Lacticin 3147 displays hydrophobic characteristics in solution and an affinity to interact with the lipids and bile salts present in gastrointestinal (GI) fluid. This could be advantageous for its development into a topical lipid-based therapy**,** increasing its solubility and its permeability into the dermis and epidermis layers of the skin when treating *S. aureus* infections locally.

For oral administration, it was observed that the endogenous components of the GI tract enhanced the solubility of both Ltnα and Ltnβ. This is promising as *L. monocytogenes* and *C. difficile* infections in the colon are potential targets for lacticin 3147. Lacticin 3147, however, is susceptible to enzyme degradation by trypsin and α-chymotrypsin in the lower digestive tract. Thus, to enable the lacticin 3147 peptides to act in the colon, they would need to be formulated in a way that protects them from this degradation. The degradation of lacticin 3147 by digestive enzymes could be overcome by nanocarrier based technology thus improving oral delivery efficiency. Amongst all nanocarriers, SLNs are considered to be superior for protection of actives against hostile GI environment by encapsulating actives in a solid matrix [38]. The increase in the solution concentration of lacticin 3147 caused by the presence of phospholipids and bile salts in FaSSGF/FaSSIF also indicates lipid systems as a potential suitable formulation strategy for lacticin 3147. Additionally, SLNs can be produced on a large scale [26], are highly stable, and can improve the bioavailability of a drug [39]. They also show excellent biocompatibility, thus demonstrating wide acceptability by the human body [38, 40]. Thus, lacticin 3147 peptides were encapsulated in SLNs and their ability to protect Ltnα and Ltnβ from digestive enzymes was investigated.

**Scheme 2:**
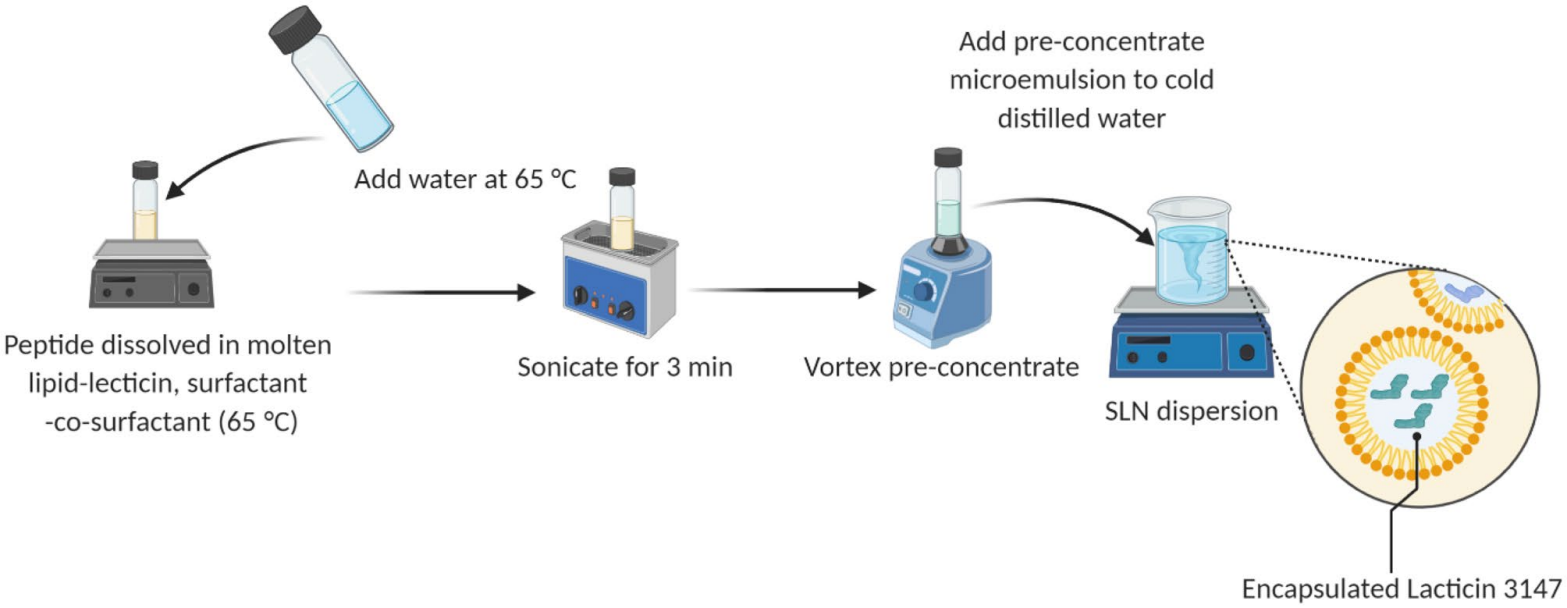
Method used to prepare solid lipid nanoparticles of lacticin 3147

Preliminary studies revealed better solubility of lacticin 3147 in geleol compared to other lipids (softisan 601 and glycerol monostearate) as determined by visual observation; hence, this was used for further studies. Geleol has the melting point between 54 and 64 °C, therefore was melted at 65 °C and maintained at this temperature until the final dilution step. As lacticin 3147 is heat stable, its exposure to such a temperature should not affect its activity [9]. In the present work, an SLN preparation was carried out using a microemulsion templating technique whose composition was derived by plotting pseudoternary phase diagrams (data not shown). This technique does not require the use of any organic solvents or post-processing steps like probe sonication or homogenization [40]. Lecithin was incorporated into the lipid matrix as an additional stabilizer. To further reduce the interfacial tension and to impart stabilization on the SLNs, a cosurfactant was used together with the surfactants [41]. Several trials were conducted by varying the surfactant type (Kolliphore® H515, Kolliphore® RH40, Labrasol and polysorbate 80, and at a 2:1 ratio with the co-surfactant Transcutol® P) and the concentration of surfactant (0.015–0.05 mg/ml) and lipid (0.005–0.01 mg/ml). Amongst all, Kolliphore® RH:40-Transcutol P (2:1) at 0.042 mg/ml and Geleol-10% lecithin at 0.0092 mg/ml gave stable solid lipid nanoparticles (Scheme 2).

The particle sizes obtained for Ltnα, Ltnβ, and blank SLNs, irrespective of the exact mass of peptide added, were 85 ± 3 nm (PDI: 0.22 ± 0.01), 81 ± 8 nm (PDI: 0.21 ± 0.01), and 82 ± 15 nm (PDI: 0.22) respectively. This demonstrates that there was no significant difference in the size of the SLNs in any of the batches with or without peptides. Similarly, the encapsulation of the bacteriocin nisin Z into nano micelles by Sadiq et al. did not cause a significant increase in size [42]. The particle sizes obtained here are similar to that reported in the literature for local delivery to the colon [43, 44]. The narrow PDIs of these dispersions indicate homogeneous SLNs of uniform size. The encapsulation of Ltnα and Ltnβ increased the zeta potential of the SLNs by + 4.6 ± 0.9 and + 3.6 ± 0.3 respectively. This value was irrespective of the mass of each peptide added in the range of peptide loadings used, 3–6 mg. The increase of the zeta potential indicates that there must be some interaction between the peptides and the SLN surface composition.

The encapsulation efficiency of Ltnα was 16 ± 6%, while the encapsulation efficiency of Ltnβ was 84 ± 8%. Ltnβ has previously shown to be the more hydrophobic peptide where the presence of micelles in FaSSIF led to a greater increase in solubility for Ltnβ than Ltnα. Thus, it is not surprising that a higher encapsulation efficiency was obtained for Ltnβ into the hydrophobic geleol SLNs.

**Fig. 10:**
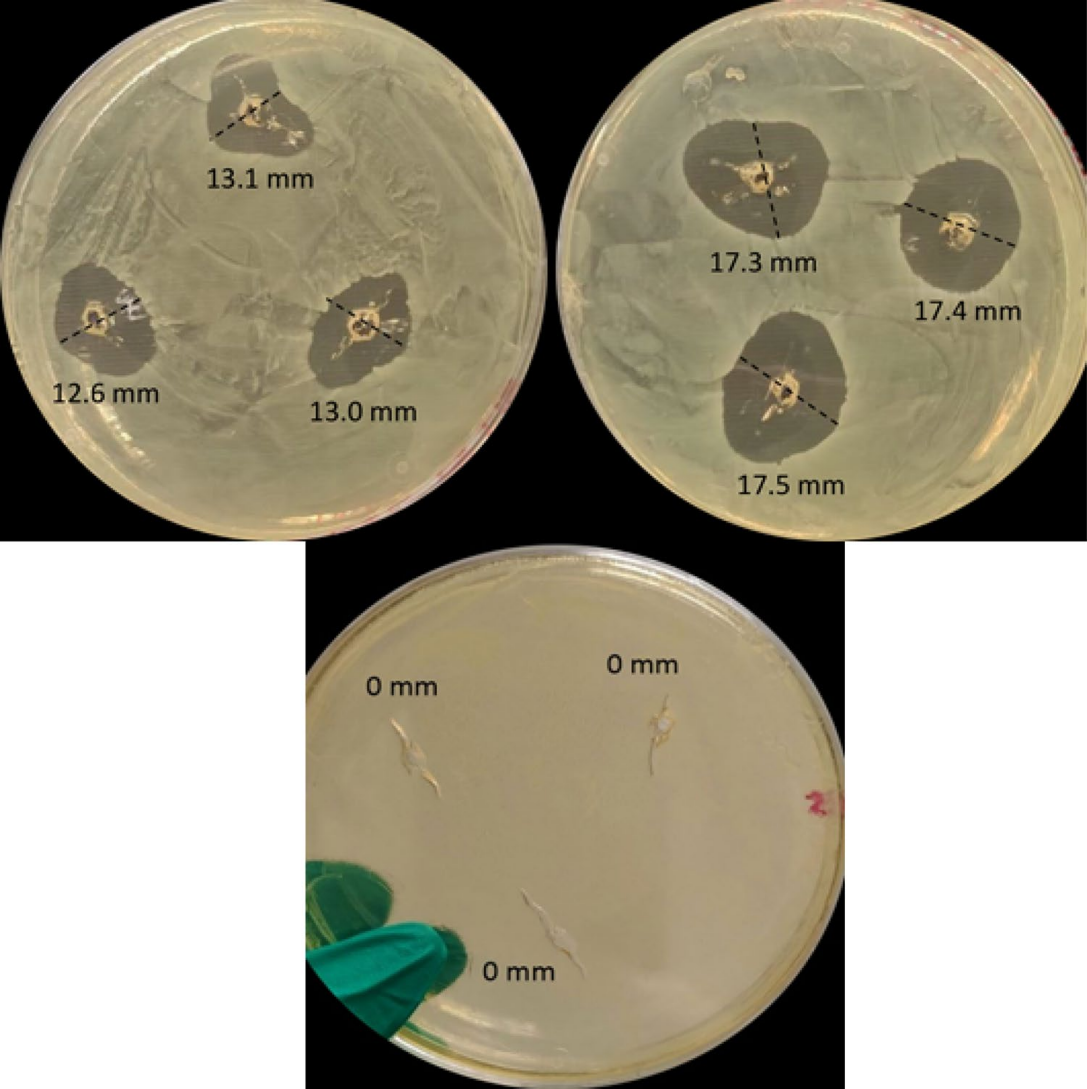
The zones of inhibition after the addition of combined free Ltnα and Ltnβ solutions (left), combined α-SLN and β-SLN dispersions (right), and blank SLN dispersions (bottom) against *L. monocytogenes* ATCC 1916. All samples were filtered before addition to the wells (*n* = 2)

An in vitro well diffusion assay (WDA) was performed to evaluate the activity of encapsulated peptide in SLNs versus free peptides (Fig. 10). A WDA was chosen instead of growth curve assays previously used for the previous activity studies as complete killing was seen for both solutions when tested by growth curve assays. The WDA method allowed for the extent of killing by each to be compared. The free peptides were suspended in water at the same concentration as the total peptide content determined for the lacticin 3147 SLNs used in the assay. The free peptide suspensions were vortexed briefly, stirred for 5 min at 1100 rpm, and then filtered to remove any undissolved lacticin 3147. They were filtered again before addition to the agar plate to ensure sterility.

The larger zone of inhibition observed with the lacticin 3147 SLNs, 17.6 ± 0.5 mm, compared to those observed with free lacticin 3147, 12.9 ± 0.3 mm, indicates (i) the retention of activity of Ltnα and Ltnβ post encapsulation and (ii) an increase in antimicrobial activity in water due to the encapsulation into SLNs. The amount of free peptide added to water in Fig. 10 (left) was the same as the total peptide content of the SLNs in Fig. 10 (right). Both the free lacticin 3147 peptide solutions and the α-SLN and β-SLN suspensions were filtered before addition to the plate to remove any undissolved lacticin 3147 and ensuring sterility. Thus, the encapsulated peptides account for the larger inhibition zone observed, i.e. greater activity is seen in the α-SLN and β-SLN plate compared to the aqueous solution of free Ltnα and Ltnβ. No zone of inhibition was seen for the blank SLNs (Fig. 10 (bottom)), indicating that all killing observed in the lacticin 3147 samples were due to lacticin 3147 only and not the components of the SLNs.

**Fig. 11:**
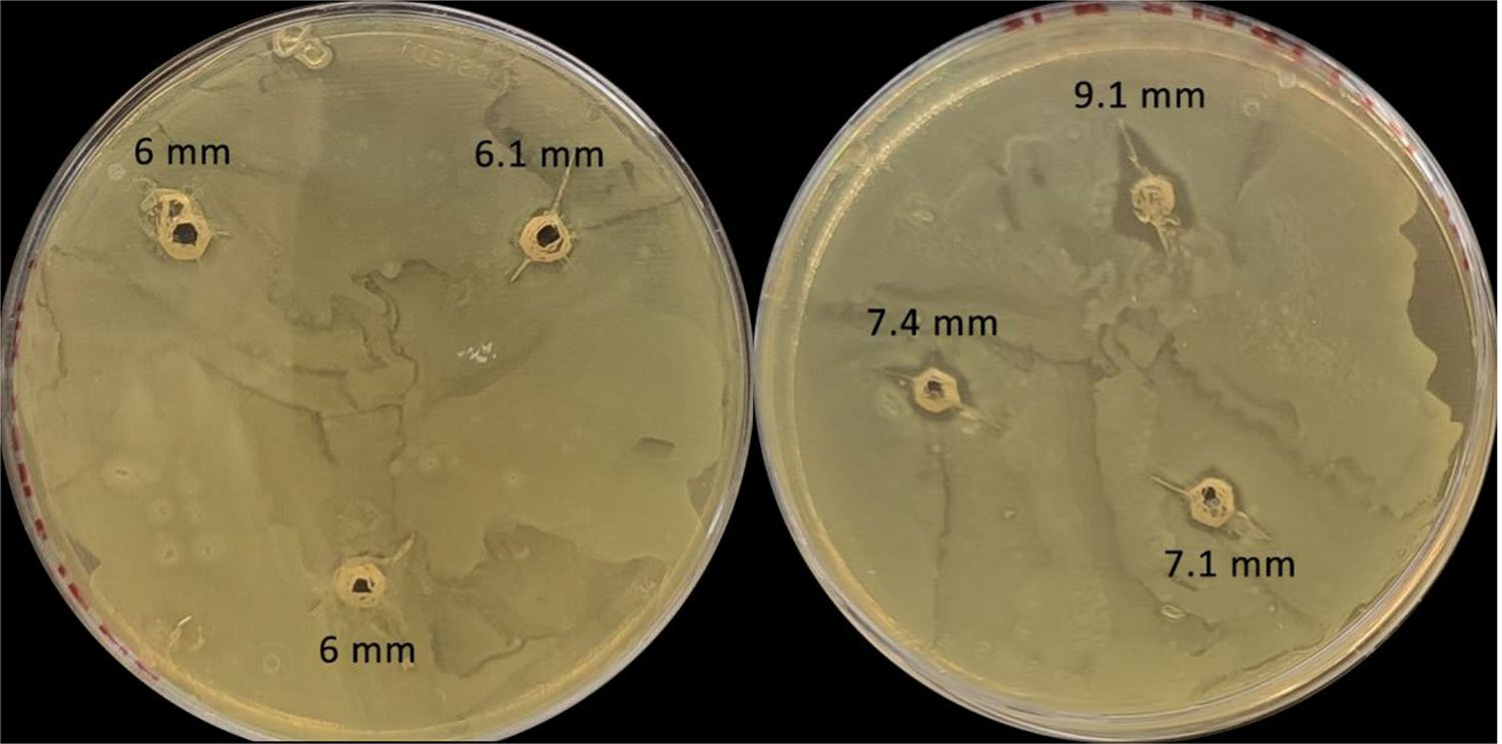
The zones of inhibition after the addition of free lacticin 3147 (left) and lacticin 3147 SLNs (right) after incubation at 1:1 with 50 mg/ml α-chymotrypsin for 3 h (*n* = 2) against *L. monocytogenes* ATCC 1916

As previously determined, Ltnα and Ltnβ are fully degraded by α-chymotrypsin, leading to the loss of their activity. To determine if the encapsulation of the lacticin 3147 peptides into SLNs can protect them from enzymatic degradation, both free Ltnα and Ltnβ and α-SLN and β-SLN were incubated with α-chymotrypsin and then tested for activity (Fig. 11).

Small, yet significant, distinct zones of inhibition were present around the lacticin 3147 SLNs after incubation with α-chymotrypsin (Fig. 11 (left)), of size 7.4 ± 0.9 mm, compared to barely visible zones with the free peptides incubated with the same, 6.0 ± 0.1 mm (Fig. 11 (right)). This indicates the ability of the SLNs to protect lacticin 3147 from degradation by this enzyme to some extent. It is important to note, however, that α-chymotrypsin is still active during the 24-h incubation with *L. monocytogenes*. This means that although lacticin 3147 was protected by the SLNs during the 3-h incubation with α-chymotrypsin, some of the peptides are likely to have been degraded when released out of the SLNs while in the wells of the agar plate. The zones of inhibition present are caused by the lacticin 3147 that survived degradation throughout the 24-h incubation (as the enzyme was not deactivated during this incubation period), meaning that the protective effect of the SLNs seen here may be less than what would be seen after only 3 h of incubation with α-chymotrypsin. Further studies are required to determine the exact level of protection they provide.

Successful encapsulation and retention of activity of Ltnα and Ltnβ indicate the feasibility of SLNs as a delivery system of lacticin 3147. The ability of the SLNs to protect the lacticin 3147 peptides from degradation by α-chymotrypsin indicates their potential for delivering intact lacticin 3147 to treat infections in the intestine or colon.

## Conclusion

Lacticin 3147 was shown to have low and unstable aqueous solution concentrations over a range of pH solutions from pH 1.6 to pH 7.4, despite demonstrating activity at such pH values. This activity is due to lacticin 3147′s potency and low MIC values. A bioactive’s physicochemical properties can suggest potential delivery strategies early on that may enable its success at later stages of development. Lacticin 3147′s poor aqueous solubility and susceptibility to proteolytic degradation will necessitate solubilization and encapsulation strategies for its success in vivo. The increase in the solution concentration of lacticin 3147 peptides caused by the presence of phospholipid molecules and NaTc surfactant molecules in biorelevant media FASSIF indicated that lipid systems could be a potential formulation strategy. Ltnα and Ltnβ retained activity when encapsulated into solid lipid nanoparticles and demonstrated enhanced activity compared to free lacticin 3147. Lacticin 3147 SLNs also showed activity after incubation with α-chymotrypsin, displaying the potential ability of SLNs’ to protect the lacticin 3147 peptides from degradation by this enzyme. Further optimization of such lipid delivery systems could deliver intact lacticin 3147 successfully to the colon to fight bacterial infections or indeed could increase the permeability of a lacticin 3147 topical treatment for infected wounds, enabling its development as an alternative to existing antibiotics.

## Data Availability

All data and material is available on request from the corresponding author.
